# Machine Learning-Based Severity Stratification for Smart Preventive Decision Support: Evidence from Measles Surveillance in a Resource-Constrained Region

**DOI:** 10.3390/jcm15103757

**Published:** 2026-05-14

**Authors:** Andrei-Florentin Baiașu, Venera-Cristina Dinescu, Cătălina-Elena Bică, Alexandra-Daniela Rotaru-Zăvăleanu, Ana-Maria Boldea, Ramona-Constantina Vasile, Mircea-Sebastian Șerbănescu, Ruxandra-Mădălina Florescu

**Affiliations:** 1 Doctoral School, University of Medicine and Pharmacy of Craiova, 2–4 Petru Rares Str., 200349 Craiova, Romania; andrei.baiasu@umfcv.ro (A.-F.B.); ruxandra.florescu@umfcv.ro (R.-M.F.); 2 Department of Health Promotion, University of Medicine and Pharmacy of Craiova, 2–4 Petru Rares Str., 200349 Craiova, Romania; venera.dinescu@umfcv.ro; 3 Department of Pediatrics, University of Medicine and Pharmacy of Craiova, 2–4 Petru Rares Str., 200349 Craiova, Romania; catalina.bica@umfcv.ro; 4 Department of Epidemiology, University of Medicine and Pharmacy of Craiova, 2–4 Petru Rares Str., 200349 Craiova, Romania; alexandra.rotaru@umfcv.ro; 5 Department of Medical Informatics and Biostatistics, University of Medicine and Pharmacy of Craiova, 2–4 Petru Rares Str., 200349 Craiova, Romania; mircea.serbanescu@umfcv.ro

**Keywords:** measles, machine learning, random forest, disease severity, vaccination coverage, surveillance, South-West Romania, public health, socioeconomic determinants, healthcare access

## Abstract

**Background/Objectives:** Vaccine-preventable diseases remain a persistent public health challenge in regions characterized by structural vulnerabilities, including suboptimal vaccination coverage, socioeconomic deprivation, and limited access to healthcare. In structurally vulnerable regions, such as the South-West Romanian region, characterized by persistent vaccination gaps and recurrent outbreaks, these conditions generate a sustained public health burden that requires ongoing preventive risk management strategies. In such contexts, digital risk stratification tools may support preventive decision-making by enabling early identification of patients at increased risk of severe outcomes. This study applied machine learning techniques to routinely collected measles surveillance data from South-West Romania to identify severe disease cases and determine key predictors of severity, offering a pragmatic alternative to outbreak forecasting in a resource-constrained setting. **Methods:** An open epidemiological dataset of laboratory-confirmed measles cases reported by the Regional Center for Public Health Surveillance Craiova was analyzed. The dataset defined severe cases as those with pneumonia, thrombocytopenia, a hospital stay exceeding three days, or other documented complications requiring medical intervention. Random Forest (RF) and Logistic Regression (LR) classifiers were trained and compared using a 10-fold cross-validation framework across 200 resampling iterations. Model performance was assessed using accuracy, AUC-ROC, sensitivity, specificity, positive predictive value, and F1-score. Feature importance was quantified using permutation-based measures, and the highest-ranked predictors were further evaluated through chi-square tests of independence. **Results:** RF significantly outperformed LR in accuracy (0.84 vs. 0.82), AUC (0.87 vs. 0.80), specificity (0.87 vs. 0.84), positive predictive value (0.89 vs. 0.86), and F1-score (0.84 vs. 0.83), with *p* ≤ 0.001 for most metrics. Sensitivity was equivalent between models (approximately 0.81; *p* = 0.328). Feature importance analysis identified seven key predictors: county of residence, vaccination status, outbreak status, presence of other symptoms, occupation, cough, and conjunctivitis. All seven were significantly associated with disease severity, and six showed significant geographic variation across counties. Vâlcea County had the highest concentration of severe cases. The model was trained on a regional surveillance cohort in which symptomatic and hospitalized cases are over-represented and should be interpreted as a triage-support tool within this surveillance context rather than as a population-level severity estimator. **Conclusions:** Machine learning, particularly RF, can effectively identify severe measles cases using routinely collected surveillance data in settings where robust outbreak prediction is not feasible. The county of residence functioned as a composite proxy for structural determinants, including healthcare access, vaccination coverage, and socioeconomic deprivation. These findings support the use of ML-based severity classification as a pragmatic tool for clinical risk stratification and targeted public health intervention in resource-constrained environments.

## 1. Introduction

Measles remains one of the most contagious vaccine-preventable diseases and a persistent public health challenge worldwide [1]. Despite an effective vaccine, outbreaks continue across Europe due to gaps in immunization, healthcare access, and surveillance [2]. Romania has been disproportionately affected over the past decade, with recurrent epidemics exposing structural vulnerabilities in both prevention and monitoring [3]. In 2024, the European region reported its highest measles case count in more than 25 years, and Romania ranked among the hardest-hit countries [4]. Outbreaks have predominantly involved unvaccinated or incompletely vaccinated individuals [5], raising concerns about whether existing surveillance and response mechanisms are adequate [6,7].

Achieving and maintaining at least 95% coverage with two doses of measles-containing vaccine is essential to interrupt transmission [8]. In Romania, where measles vaccination is recommended but not mandatory [9,10,11], coverage declined from 97% in 2005 to 86% in 2016 [12], reaching a nadir of 83.4% for the first dose and 71.4% for the second in 2022, with substantial inter-county variation [6]. Comparable European countries that introduced or retained mandatory vaccination have generally maintained higher coverage [13]. Supplementary immunization activities are recommended during outbreaks [14], but reported national coverage often overestimates true protection because of missed doses, delayed schedules, incomplete records, and clusters of under-vaccinated communities [15].

Socioeconomic conditions further compound vulnerability. Lower educational attainment is consistently linked to reduced vaccine acceptance, limited health literacy, and delayed recognition of disease severity [16,17,18], and the South-West Oltenia region is characterized by low tertiary education, high early school leaving, and elevated rates of poverty and material deprivation [19,20,21,22]. Financial constraints, overcrowded living conditions, and poor nutrition increase baseline susceptibility and may worsen clinical presentations [23,24], and international evidence confirms that measles case-fatality is shaped by maternal education, GDP per capita, and urbanization [25].

Access to primary healthcare is also unevenly distributed in Romania, with severe shortages in rural and underserved areas [26,27,28]: 328 of 3181 local governments lack any family physician, amounting to a national deficit of approximately 2200, with most physicians and nurses concentrated in urban settings [29,30]. The absence of school-entry vaccination requirements compounds these disparities and reduces opportunities for routine immunization, early diagnosis, and timely follow-up [31]. Limited primary care access has been associated with delayed presentation, more advanced disease at diagnosis, and higher rates of complications [32,33]. In Western Romanian populations specifically, risk factors for prolonged hospitalization include chronic lung disease, liver damage, Roma ethnicity, extended intervals since the last MMR dose, elevated inflammatory markers, and radiological evidence of pulmonary involvement [34], with hospitalized patients generally representing the more severe end of the clinical spectrum [35]. At the administrative level, Public Health Directorates (Direcția de Sănătate Publică, lb. rom—DSP) serve as the primary structures for county-level surveillance, but reporting delays, data fragmentation, and limited regional integration constrain their utility for real-time outbreak prediction.

Against this backdrop, artificial intelligence and machine learning have attracted growing attention in infectious disease surveillance, with potential for outbreak prediction, early warning, and public health planning [36,37]. Algorithms such as Random Forest, Support Vector Machines, neural networks, and deep learning architectures have been used to forecast disease incidence and identify high-risk populations across various epidemiological contexts [38]. Their effectiveness, however, depends on large, high-quality, well-structured datasets [39]. In Romania, as in many settings with suboptimal surveillance, infectious disease databases are fragmented, inconsistently structured, and designed primarily for mandatory reporting rather than predictive modeling [40], with data acquisition delays [41] and limited cross-regional integration [42] further constraining feasibility. Additional concerns include data quality and bias, model transparency, system integration, and ethical issues around privacy and equity [43].

Where robust outbreak prediction is not yet feasible, alternative applications of ML may still offer meaningful value [44]. One such application is the identification of severe disease presentations among confirmed cases, an approach less dependent on comprehensive population-level data [45] and aligned with the recognition that forecasts only translate into effective responses when embedded in decision-support systems that guide clinical action and resource allocation [46]. By modeling demographic, vaccination-related, socioeconomic, and clinical variables against markers of severity such as hospitalization and prolonged length of stay, ML can support early identification of high-risk patients [47]. Established risk factors for severe measles include extremes of age, lack of vaccination, immunodeficiency, pregnancy, malnutrition, crowding, and vitamin A deficiency [48], and the inclusion of clinical features and complications can further improve discrimination between severe and uncomplicated cases [49]. Such a strategy has the potential to improve clinical decision-making and optimize resource allocation [50], while identifying modifiable risk factors that can inform targeted public health interventions [51].

The present study applies machine learning techniques to routinely collected measles surveillance data from South-West Romania, aiming to identify severe disease cases in a setting with limited outbreak surveillance capacity. Rather than focusing on transmission dynamics or outbreak forecasting, the emphasis is on severity classification, a clinically meaningful and operationally feasible application of ML within a resource-constrained public health environment. A secondary objective is to perform a comprehensive statistical analysis of the dataset to facilitate interpretation of model outputs and to identify variables associated with severe measles that may support future surveillance and planning efforts.

The novelty of this work lies not in proposing a new algorithm but in developing a clinically and operationally oriented severity stratification framework using routinely collected surveillance data from a structurally vulnerable, resource-constrained region. In contrast to most prior ML studies in infectious diseases, which focus on incidence forecasting under richer data conditions, this study addresses early identification of severe cases when robust real-time forecasting is not feasible, using an operational severity definition adapted to a real-world regional dataset and a hybrid analytical strategy that integrates ML-based feature prioritization with classical inferential statistics for interpretability and public-health relevance.

## 2. Materials and Methods

### 2.1. Study Design

The analysis was performed using an open epidemiological dataset of 624 laboratory-confirmed measles cases reported at the regional level by the Public Health Surveillance Craiova (Centrul Regional de Sănătate Publică Craiova), which serves the South-West region of Romania, during 2023–2024. The dataset was compiled as part of routine surveillance activities and includes standardized case records collected during outbreak monitoring. It comprised a broad set of demographics, clinical, and epidemiological variables relevant to measles severity modeling (27 variables); from this set, a subset was selected based on clinical relevance, data completeness, and applicability to severity prediction. The selected variables are summarized in Table 1, grouped by category.

Severe-case status was available as a predefined field in the regional surveillance dataset and was not computed. According to the dataset documentation, this variable followed the definition provided in [1]. Dataset-defined severe cases were reported as being associated with one or more recorded clinical indicators, including radiologically or clinically confirmed pneumonia, thrombocytopenia, a length of hospital stay exceeding three days, or complications requiring medical intervention, such as laryngotracheobronchitis/croup, otitis media, keratitis, conjunctivitis, stomatitis, or dehydration. However, the dataset did not provide case-level details indicating which specific criterion or combination of criteria determined the severe-case status for each individual patient.

During data review, we compared the available severity-related fields with broader World Health Organization (WHO) criteria for severe measles, which include severe diarrhea with dehydration, acute encephalitis, acute disseminated encephalomyelitis, subacute sclerosing panencephalitis, severe respiratory distress, hemorrhagic measles, and blindness [52]. WHO and CDC guidelines further indicate that measles can result in complications such as diarrhea, dehydration, pneumonia, encephalitis, and death, with approximately 20% of cases requiring hospitalization in high-income settings and up to 30% experiencing at least one complication [53].

However, several WHO-listed manifestations, including diarrhea, encephalitis, acute disseminated encephalomyelitis, subacute sclerosing panencephalitis, and other severe neurological or gastrointestinal manifestations, were not documented as informative variables in the dataset, either because they were not available as separate fields or because they showed no recorded variation. Because it was not possible to determine whether their absence reflected true epidemiological absence, underreporting, or limitations in data collection, these variables were treated as unobserved or non-informative in the available dataset rather than as confirmed absent clinical events. Consequently, we did not reinterpret, expand, or recompute the predefined severity label using these criteria. Instead, we retained the dataset-provided severe-case variable as the study outcome and interpreted it as a pragmatic surveillance-based severity classification, consistent with recommendations for adapting severity classifications to the data infrastructure available in resource-constrained surveillance settings [54].

### 2.2. Variable Selection

Data quality was systematically assessed prior to model development. All records were screened for missing values, non-informative variables with no variance, and extreme values. The initial surveillance database comprised 624 laboratory-confirmed measles cases described by 54 fields. Record identifiers and administrative fields, such as unique keys, record numbers, notification dates, and onset dates; variables exhibiting no variability across cases; and fields used to define the severity outcome were excluded to avoid information leakage, bias, or model instability. The final analytical dataset retained all 624 cases with 27 predictor variables (Table 1). No imputation was required, and no cases were dropped due to missing data. Categorical variables were encoded using schemes compatible with the applied algorithms, and continuous variables were standardized to ensure comparability across predictors and to prevent scale-dependent dominance during training. The dataset was then partitioned using a cross-validation framework, which minimizes the risk of overfitting by evaluating model performance on data not used during training and provides more reliable estimates than a single train–test split, particularly in datasets of moderate size [55].

### 2.3. Machine Learning Models

Several supervised machine learning classification models were initially implemented to predict severe measles cases. These models were selected to represent both conventional statistical approaches and ensemble-based learning methods, including Logistic Regression (LR), Random Forest (RF), Random Subspace (RS), and Regularized Ensemble (RE) models.

LR was included as a baseline parametric model because it provides a transparent and interpretable framework for binary classification, which is particularly useful in medical contexts where understanding the direction and magnitude of predictor effects is important. RF was included as a flexible ensemble method capable of capturing non-linear relationships and complex interactions, with good robustness to overfitting and suitability for mixed variable types [56]. RS, also known as attribute bagging, was included to test whether training multiple classifiers on random subsets of predictors could improve performance in the presence of potentially correlated variables. RE models were also evaluated to assess whether combining multiple learners while controlling model complexity could further improve predictive accuracy.

All candidate models were first compared using classification accuracy as a screening metric. Based on this initial comparison, RF showed the best overall predictive performance and was therefore selected as the main model for subsequent analyses. LR was retained as the comparator model because it was the best-performing interpretable baseline and allowed direct comparison between a flexible ensemble-based approach and a traditional parametric method. The other models, RS and RE methods, showed lower performance in the screening phase and were therefore not carried forward into the detailed comparative analysis.

**Figure 1:**
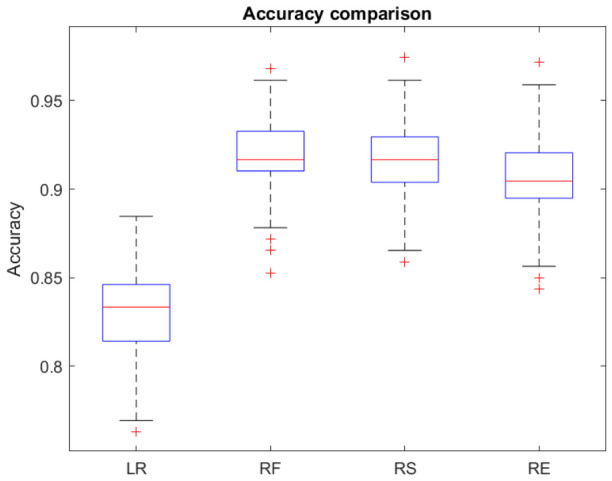
Initial screening of candidate machine learning models for severe measles classification. Classification accuracy was used as the primary screening metric to compare the performance of the candidate algorithms, including LR, RF, RS, and RE models. RF showed the highest predictive performance and was therefore selected as the main model for subsequent analysis, while LR was retained as the interpretable baseline comparator.

Accordingly, the final model evaluation focused on RF and LR. These two models were subjected to formal statistical performance comparisons using the same data partitions and evaluation metrics. RF was also used for feature-importance analysis to identify the demographic, clinical, and epidemiological variables most strongly contributing to severe-case classification (Figure 1).

### 2.4. Formal Mathematical Description of the Prediction Models

For each case i, let y_i_ ∈ {0, 1} denote the outcome label, where y_i_ = 1 corresponds to a severe case and y_i_ = 0 to a non-severe case. Let x_i_ = (x_i1_, x_i2_, …, x_i_p)^T^ denote the vector of predictor variables for observation i.

Logistic regression models the conditional probability that a case belongs to the severe class as a function of the predictor vector. The probability π_i_ of severe disease for case i is defined as:

P(y_i_ = 1|x_i_) = π_i_ = 1/[1 + exp(−(β_0_ + Σ_j = 1_^p^ β_j_ x_ij_))] (1)

Equivalently, the model can be expressed in logit form as:

log(π_i_/(1 − π_i_)) = β_0_ + Σ_j = 1_^p^ β_j_ x_ij_ (2)

where β_0_ is the intercept and β_j_ represents the regression coefficient associated with predictor j. Model coefficients are estimated by maximum likelihood, and the fitted model yields an estimated probability for each case, which can then be thresholded for binary classification.

Random Forest is an ensemble learning method composed of B decision trees, each trained on a bootstrap sample of the original dataset. For a given observation x_i_, each tree T_b(x_i_) produces a class prediction in {0, 1}. The final class prediction is obtained by majority voting:

ŷ_i_ = mode{T_1_(x_i_), T_2_(x_i_), …, T^B^(x_i_)} (3)

The estimated probability that case i belongs to the severe class can be expressed as the fraction of trees voting for class 1:

P(y_i_ = 1|x_i_) = (1/B) Σ_β = 1_^B^ I(T_b(x_i_) = 1) (4)

where I(·) denotes the indicator function, equal to 1 when the condition is true and 0 otherwise. At each split within each tree, only a random subset of predictors is considered, which reduces correlation among trees and improves generalization performance.

To assess the contribution of each predictor within the Random Forest model, permutation importance can be computed by measuring the decrease in model performance after randomly permuting the values of predictor X_j_:

I_j_ = M_baseline − M_perm(j) (5)

where M_baseline denotes the baseline value of a chosen performance metric and M_perm(j) denotes the same metric after permutation of predictor X_j_. A larger value of I_j_ indicates a greater contribution of predictor X_j_ to model performance.

### 2.5. Performance Metrics and Statistical Analysis

Beyond accuracy, performance was assessed using a comprehensive set of metrics appropriate for binary classification in medical settings: sensitivity (recall), specificity, positive predictive value (precision), area under the receiver operating characteristic curve (AUC-ROC), and F1-score.

Evaluation was conducted using a repeated non-stratified 10-fold cross-validation framework to ensure robustness and reduce the risk of overfitting. In each iteration, the dataset was randomly partitioned into ten approximately equal folds, without enforcing class-stratified sampling. Nine folds were used for model training, while the remaining fold was held out for testing. This process was repeated ten times so that each fold served once as the test set. Performance metrics were calculated separately for each held-out fold and then averaged to obtain one cross-validation estimate per iteration. The entire non-stratified 10-fold cross-validation procedure was repeated across 200 resampling iterations using different random partitions, and the final results were reported as mean ± standard deviation across these repeated evaluations.

For each binary classification task, predictions were summarized using a confusion matrix, which reports true positives (TP), true negatives (TN), false positives (FP), and false negatives (FN). From these, accuracy, sensitivity, specificity, positive and negative predictive values, F1-score, and odds ratio were computed according to standard formulations summarized in Table 2. The threshold-independent ROC/AUC framework, complementary to these discrete metrics, is defined in Equations (6)–(8) below. ROC curves were generated to assess discriminative ability across a range of classification thresholds, plotting the true positive rate against the false positive rate, with the AUC providing a threshold-independent summary measure ranging from 0.5 (no discrimination) to 1.0 (perfect discrimination). The ROC curve is obtained by plotting the true positive rate (TPR) against the false positive rate (FPR) over a range of classification thresholds. The true positive rate and false positive rate are defined as:

TPR = TP/(TP + FN)(6)

FPR = FP/(FP + TN)(7)

The AUC provides a threshold-independent measure of discriminative performance and may be expressed as:

AUC = ∫_0_^1^ TPR(FPR^−1^(u)) du (8)

Paired comparisons between the RF and LR models were performed to evaluate differences in predictive performance across identical test splits. For each metric, paired Student’s *t*-tests were initially applied to the paired performance estimates obtained from matched resampling iterations. This approach was considered appropriate because the large number of repeated evaluations allowed the distribution of the mean paired differences to be approximated under the Central Limit Theorem.

However, because repeated resampling can introduce dependence between estimates and may partially violate the independence assumptions of conventional parametric tests, the analyses were also repeated using the Wilcoxon signed-rank test for paired samples. The Wilcoxon analysis yielded the same statistical interpretation as the paired *t*-test for all evaluated metrics. Accordingly, paired *t*-test results are reported in the main tables to remain consistent with the mean ± standard deviation presentation of model performance, while the Wilcoxon signed-rank test was used as a robustness check. All tests were two-tailed, with statistical significance set at *p* < 0.05.

Following the identification of the most influential predictors by the RF algorithm, a complementary classical statistical analysis was carried out to further characterize their association with the severe case definition. The highest-ranked features were evaluated using descriptive methods and visualized through graphical representations to explore distributional patterns and group differences. For categorical variables, associations with severity were formally assessed using the chi-square (χ^2^) test of independence, with significance defined as *p* < 0.05. This combined approach, machine learning–driven feature selection paired with traditional inferential statistics, enabled both data-driven prioritization of predictors and clinically interpretable validation of their relevance.

Algorithm implementation, model training, and performance evaluation were carried out in MATLAB (version 9.10 (R2021a), MathWorks, Natick, MA, USA). Statistical analyses, result aggregation, and data management were performed in MATLAB, with Microsoft Excel-based workflows for supplementary analysis, tabulation, and visualization. The study was conducted as a retrospective analysis of a routinely collected, open, anonymized measles surveillance dataset. The research protocol was reviewed and approved by the Ethics Committee for University and Scientific Deontology (Comisia de Etică și Deontologie Universitară și Științifică) of the University of Medicine and Pharmacy of Craiova (approval no. 368/3 September 2025). All procedures were carried out in accordance with the Declaration of Helsinki, the University Code of Ethics, and the Medical Deontology Code. Given the retrospective nature of the study and the use of de-identified data, the requirement for individual informed consent was waived.

The manuscript was drafted by the authors based on original research conducted and analyzed according to the methodology described below.

For language editing, stylistic refinement, and structural reorganization of author-written text during final manuscript preparation, Anthropic’s Claude (Claude Opus 4.6, accessed via claude.ai (accessed on 20 March 2026)) was employed as a writing assistance tool. The AI tool was used exclusively for grammar, spelling, and punctuation correction of author-drafted text; sentence restructuring for clarity and coherence; organizational improvement and paragraph reorganization; consistency in terminology; and readability enhancement.

All scientific content, data collection, statistical analysis, interpretation of results, study design, methodology, figures, tables, and conclusions originated entirely from the authors. The AI tool did not generate novel text or original scientific arguments or data and did not contribute to any aspect of the research design or intellectual framework. All AI-assisted edits were reviewed and approved by the authors prior to final submission. The authors retain full responsibility for the accuracy and integrity of all manuscript content.

## 3. Results

### 3.1. Dataset Characteristics

The analyzed dataset comprised 624 laboratory-confirmed measles cases reported during 2023–2024 across the five counties of the South-West Oltenia region: Dolj (DJ), Gorj (GJ), Mehedinți (MH), Olt (OT), and Vâlcea (VL). The majority of cases were reported in Vâlcea county (n = 311, 49.8%), followed by Dolj (n = 156, 25.0%), Olt (n = 75, 12.0%), Gorj (n = 65, 10.4%), and Mehedinți (n = 17, 2.7%). Of all cases, 51.8% were male and 48.2% female, with a median age of 5 years (range 2–14). Severe cases, as defined by the operational criteria described in Section 2.1, accounted for 55.0% of the total dataset (n = 343), while 45.0% were classified as non-severe (n = 281). The distribution of cases by county and severity category is summarized in Table 3.

### 3.2. Comparative Performance Analysis of RF and LR

The predictive performance of the two retained models, RF and LR, was evaluated using multiple classification metrics computed across 200 iterations. The results are presented in Table 4, together with the statistical assessment based on paired Student’s *t*-tests.

Both models demonstrated good overall discriminative ability for identifying severe measles cases. RF achieved a mean accuracy of 0.84 ± 0.04 and an AUC of 0.87 ± 0.05, while LR yielded a mean accuracy of 0.82 ± 0.05 and an AUC of 0.80 ± 0.06.

Paired statistical comparisons revealed that RF significantly outperformed LR across the majority of performance metrics. Statistically significant differences (*p* < 0.001) were observed for accuracy, AUC, specificity, PPV, and F1-score. The difference in NPV was also statistically significant (*p* = 0.001), although the absolute difference between models was modest (0.80 vs. 0.79).

Notably, sensitivity was equivalent between the two models, with no statistically significant difference detected (*p* = 0.328). This finding indicates that both approaches demonstrated comparable ability to correctly identify true-positive cases (i.e., patients with severe measles), whereas RF exhibited superior performance in correctly classifying non-severe cases (higher specificity) and in overall positive-prediction precision (higher PPV).

The superior AUC of RF (0.87 vs. 0.80) suggests better threshold-independent discriminative capacity, reflecting the model’s enhanced ability to distinguish between severe and non-severe cases across the full range of classification thresholds.

**Figure 2:**
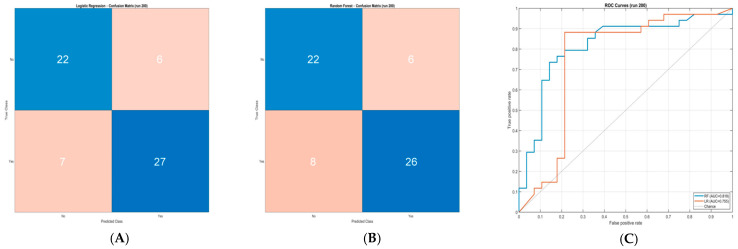
Confusion matrices and receiver operating characteristic (ROC) curves for RF (**A**) and LR (**B**) from a representative algorithm run (last). Confusion matrices display the counts of true positives (TP), true negatives (TN), false positives (FP), and false negatives (FN). ROC curves (**C**) illustrate the relationship between true positive rate (sensitivity) and false positive rate (1 − specificity) across classification thresholds, with the area under the curve (AUC) indicated.

Confusion matrices from the most recent representative run of the algorithm are presented in Figure 2, along with the corresponding receiver operating characteristic (ROC) curves, which visually illustrate the trade-off between sensitivity and specificity across decision thresholds for both models.

The comparative analysis based on aggregated results confirms the superior performance of the RF model relative to LR across most evaluated performance metrics (Table 2), and the performance values, expressed as mean ± standard deviation, are derived from repeated evaluations on identical data partitions.

### 3.3. Feature Importance Analysis

The RF model was further examined to identify the relative importance of individual predictors in classifying disease severity. Feature importance was quantified using the permutation-based importance measure derived from the RF algorithm, which reflects the decrease in model performance resulting from permuting each predictor.

**Figure 3:**
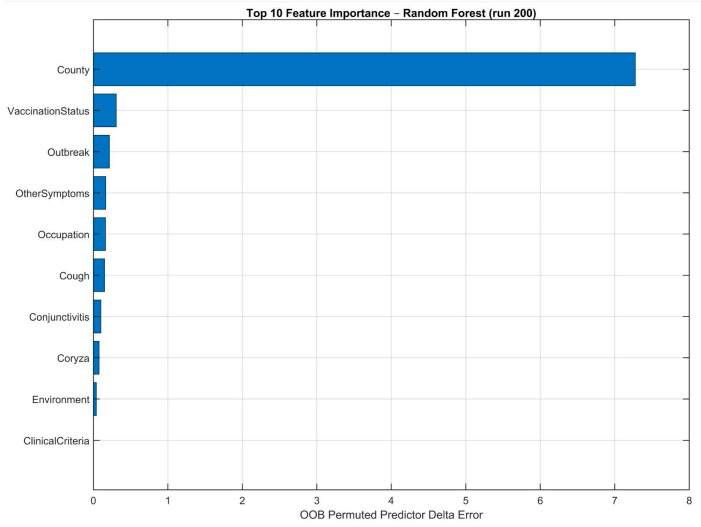
Feature importance ranking derived from the RF model. Importance scores were calculated using the permutation-based method. Higher values indicate greater contribution to model performance.

The ranked feature-importance scores are presented in Figure 3, which illustrates the relative contributions of each variable to the model’s overall predictive performance. Given the moderate size of the dataset and in order to reduce model complexity while maintaining interpretability, only the seven highest-ranked features were selected for subsequent statistical evaluation.

These top-ranked features demonstrated a clear separation in importance compared with lower-ranked variables, suggesting a dominant role in the model’s decision-making process. Restricting the analysis to the first seven features enabled focused downstream analysis while minimizing the risk of overfitting and spurious associations that may arise from including a large number of weak predictors in a limited dataset.

To assess the extent to which the predictive performance of the models was driven by the county-of-residence variable, an additional sensitivity analysis was performed. In this secondary analysis, the full modeling workflow was repeated after excluding county from the set of predictor variables. All other preprocessing steps, cross-validation procedures, resampling iterations, model-training settings, and performance metrics were kept identical to those used in the primary analysis. The purpose of this analysis was not to replace the primary model, but to evaluate the dependency of model performance on this highly informative geographic variable and to determine whether clinically and epidemiologically relevant predictive information remained after its removal.

The results of this county-excluded analysis are presented in Table 5. Removing county from the prediction variables led to a marked decrease in performance across all evaluated metrics. For RF, accuracy decreased from 0.84 ± 0.04 to 0.68 ± 0.03, and AUC decreased from 0.87 ± 0.05 to 0.73 ± 0.03. A similar decrease was observed for LR, with accuracy declining from 0.82 ± 0.05 to 0.66 ± 0.03 and AUC from 0.80 ± 0.06 to 0.68 ± 0.03. Despite this reduction, RF continued to outperform LR for most performance metrics, including accuracy, AUC, sensitivity, PPV, NPV, and F1-score. Specificity did not differ significantly between the two models in the county-excluded analysis.

Beyond aggregate performance, the county-excluded analysis also allows examination of which non-geographic variables carry independent predictive information. Feature-importance ranking of the RF model trained on the 20 non-geographic predictors identified age, occupation, conjunctivitis, vaccination status, coryza, environment (urban/rural), contact with a confirmed case, and cough as the most informative variables. The retention of moderate discriminative ability after county removal (AUC ≈ 0.73), combined with this ranking, indicates that clinical, demographic, vaccination-related, and epidemiological factors contribute meaningful predictive value independent of geographic location, while also quantifying the share of predictive information that county carries beyond these variables in the available dataset.

### 3.4. Statistical Evaluation of Key Predictors

**Figure 4:**
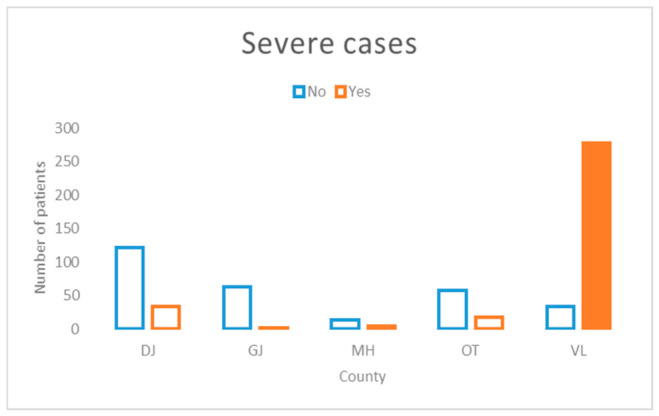
Distribution of severe measles cases stratified by county of residence. VL county (highlighted and filled) had the highest proportion of severe cases. The chi-square test confirmed a statistically significant association between county and disease severity (χ^2^ = 325.45, *p* < 0.00001).

The most prominent feature identified by the RF algorithm was the county of residence. As illustrated in Figure 4, Vâlcea County accounted for the majority of severe cases within the study population.

Given the magnitude of the importance score for the county variable, subsequent analyses focused on disease severity and were stratified by county. This approach enabled a more detailed examination of geographic variability in the distribution of severe cases and facilitated the assessment of region-specific patterns relevant to public health surveillance and intervention planning.

**Figure 5:**
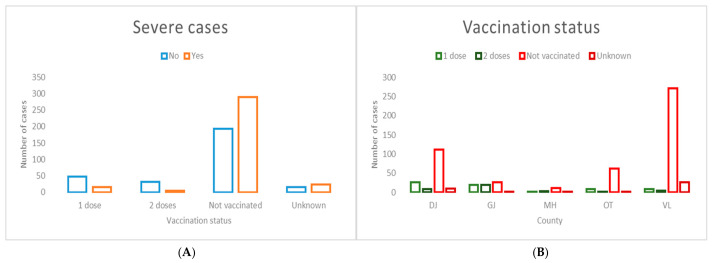
Vaccination status distribution stratified by disease severity (**A**) and county of residence (**B**). (**A**) Vaccination status was significantly associated with severe measles presentation (χ^2^ = 53.68, *p* < 0.00001). (**B**) Significant inter-county variation in vaccination status was observed across the South-West Oltenia region (χ^2^ = 143.27, *p* < 0.00001).

Vaccination status was significantly associated with severe measles cases (Figure 5). In addition, marked inter-county variation was observed, with Vâlcea (VL) County exhibiting the highest proportion of unvaccinated patients among severe cases.

**Figure 6:**
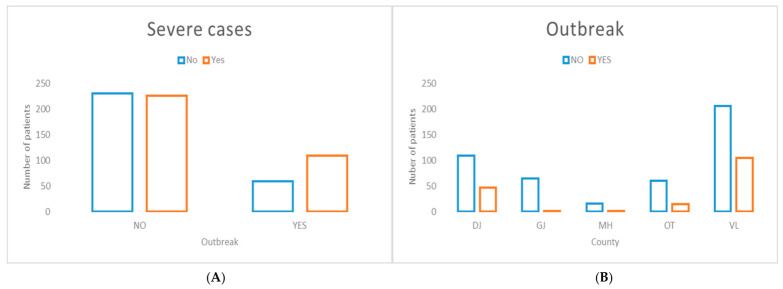
Outbreak status distribution stratified by disease severity (**A**) and county of residence (**B**). (**A**) Cases occurring within outbreak settings were significantly associated with severe disease presentation (χ^2^ = 11.59, *p* = 0.000664). (**B**) Significant inter-county variation in outbreak occurrence was observed across the South-West Oltenia region (χ^2^ = 35.01, *p* < 0.00001).

Cases occurring within outbreak settings were significantly associated with severe disease presentation (Figure 6). Although substantial inter-county variability in outbreak occurrence was observed, it was not attributable to Vâlcea County.

**Figure 7:**
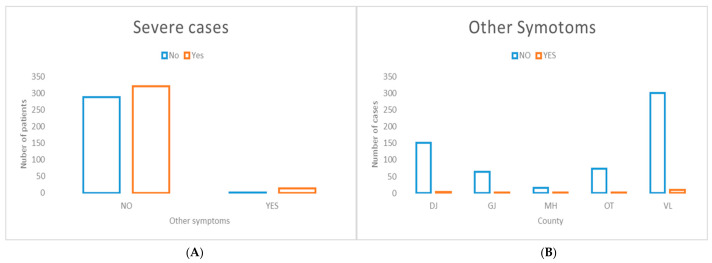
Distribution of cases presenting with other symptoms stratified by disease severity (**A**) and county of residence (**B**). (**A**) The presence of other symptoms was significantly associated with severe disease presentation (χ^2^ = 9.72, *p* = 0.001826). (**B**) No significant inter-county variation was observed in the distribution of other symptoms (χ^2^ = 1.83, *p* = 0.767).

The distribution of cases presenting with additional symptoms was significantly associated with severe disease presentation and did not exhibit significant geographic variation at the county level (Figure 7). Notably, a higher frequency of such cases was observed in DJ and VL counties, with different probable causes.

**Figure 8:**
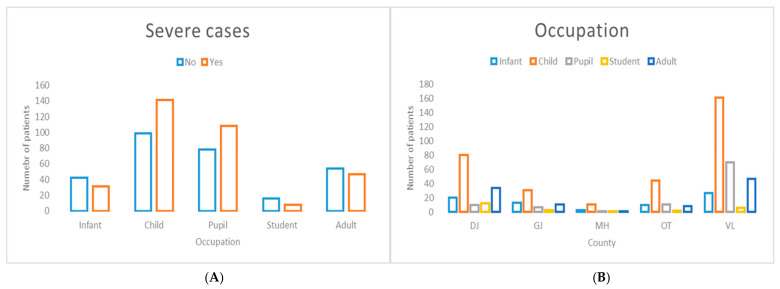
Distribution of cases by occupation stratified by disease severity (**A**) and county of residence (**B**). (**A**) Occupation was significantly associated with severe disease presentation (χ^2^ = 13.68, *p* = 0.008385). (**B**) Significant inter-county variation in occupational distribution was observed across the South-West Oltenia region (χ^2^ = 43.46, *p* = 0.000238).

Occupation was significantly associated with disease severity (Figure 8). In addition, the distribution of occupational categories differed significantly across counties, indicating geographic variability in the occupational profile of affected cases. Notably, individuals working in the education sector represented the most affected occupational group among severe cases.

**Figure 9:**
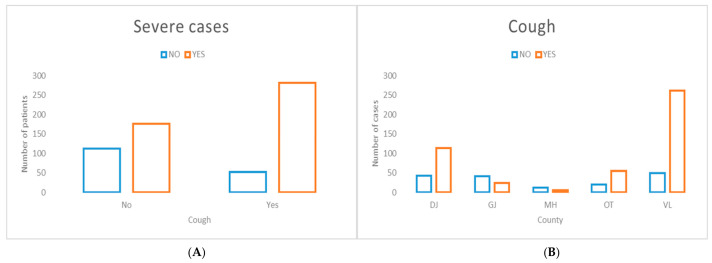
Distribution of cases presenting with cough stratified by disease severity (**A**) and county of residence (**B**). (**A**) The presence of cough was highly significantly associated with severe disease presentation (χ^2^ = 41.95, *p* < 0.00001). (**B**) Significant inter-county variation was observed in the prevalence of cough among measles cases (χ^2^ = 80.25, *p* < 0.00001).

Cough, a common respiratory symptom, was significantly associated with severe disease presentation (Figure 9), with notable inter-county variability. This geographic heterogeneity may be partially related to differences in the occupational profile of affected cases across counties, as previously observed (Figure 8).

**Figure 10:**
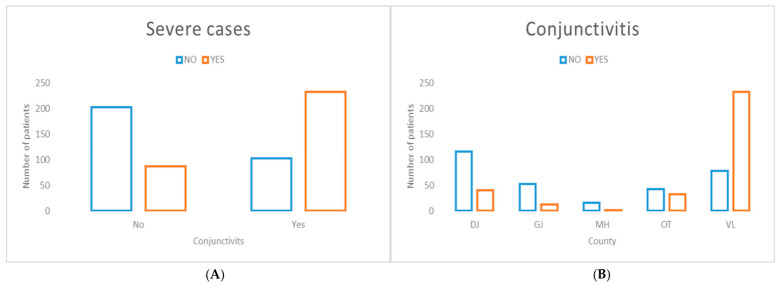
Distribution of cases presenting with conjunctivitis stratified by disease severity (**A**) and county of residence (**B**). (**A**) The presence of conjunctivitis demonstrated the strongest association with severe disease presentation among clinical symptoms evaluated (χ^2^ = 95.17, *p* < 0.00001). (**B**) Significant inter-county variation was observed in the prevalence of conjunctivitis among measles cases (χ^2^ = 151.68, *p* < 0.00001).

Conjunctivitis, a non-specific clinical symptom, demonstrated the strongest association with severe disease presentation among the evaluated clinical symptoms (Figure 10). In addition, a pronounced geographic association was observed, with a higher concentration of severe cases presenting with conjunctivitis in Vâlcea County.

## 4. Discussion

This study evaluated a machine learning-based severity stratification model designed to support preventive decision-making in a resource-constrained setting characterized by structural healthcare disparities. The measles surveillance data was collected from South-West Romania to identify severe disease in a setting characterized by limited outbreak surveillance capacity and structural healthcare disparities. The comparative analysis showed that the RF model substantially outperformed conventional LR in predicting disease severity, with consistent improvements across all evaluated performance metrics. RF achieved superior accuracy (0.84 vs. 0.82), AUC (0.87 vs. 0.80), specificity (0.87 vs. 0.84), positive predictive value (0.89 vs. 0.86), and F1-score (0.84 vs. 0.83), with statistically significant differences confirmed by paired Student’s *t*-tests (*p* ≤ 0.001 for most metrics). These results are broadly consistent with the existing literature on machine learning for predicting infectious disease severity. A systematic review of ML approaches in the MENA region reported mean accuracy scores of 0.85 for RF algorithms in disease outbreak prediction [57], and studies applying ML to COVID-19 severity have reported comparable figures, 84.5% accuracy and an AUC of 0.97 in one Chinese cohort [58], with AUC values of 0.80–0.83 for predicting intensive care needs in another [59]. In the context of measles, a study from Ghana comparing multiple ML classifiers found that RF achieved superior accuracy, precision, and recall [60]. The accuracy of approximately 84% achieved here falls within the expected range for ML-based severity classification in surveillance settings with limited data infrastructure.

The proportion of cases classified as severe in this cohort (55.0%) is substantially higher than the 20–30% complication rate typically reported in the international measles literature [53], and this discrepancy warrants explicit consideration before the model performance is interpreted. Two non-exclusive factors most plausibly account for the divergence. First, the dataset originates from a regional public-health surveillance system, which preferentially captures cases that come to medical attention. In the analyzed cohort, 74.0% of patients were hospitalized, 52.4% had documented pneumonia, and 77.4% were unvaccinated, proportions that diverge sharply from population-level estimates of approximately 20% hospitalization and 6–7% pneumonia reported in high-income settings [53]. This pattern is consistent with substantial under-ascertainment of mild, self-managed, and clinically inapparent cases and indicates that the analyzed population is shifted toward the more clinically evident end of the case spectrum. Second, the operational severity definition employed here flags the presence of any documented complication captured in the surveillance dataset, which is broader than definitions restricted to specific severe manifestations such as encephalitis or severe respiratory distress. The combination of selection toward symptomatic and hospitalized cases and an inclusive operational definition therefore offers the most parsimonious explanation for the observed proportion. Importantly, this means the reported model performance characterizes discrimination within the surveillance population, that is, among cases that have already entered the public-health surveillance system, and should not be extrapolated to estimate measles severity at the general-population level. The model is intended as a decision-support tool for prioritizing higher-risk cases among those already under surveillance, not as a population-level severity estimator.

The sensitivity analysis performed after removing county of residence from the predictor set confirmed the strong contribution of this variable to model performance. RF accuracy decreased from 0.84 to 0.68, and AUC decreased from 0.87 to 0.73, indicating that the county carried substantial predictive information.

This finding should not be interpreted as evidence that the county itself is a causal determinant of severe measles. Rather, county likely acts as a composite proxy for unmeasured structural factors, including healthcare access, vaccination coverage, socioeconomic vulnerability, rurality, health literacy, referral patterns, and possible reporting differences. In routinely collected surveillance datasets, such determinants are often unavailable at the individual level, making geographic variables indirectly informative.

Importantly, RF retained moderate discriminative ability after county removal, suggesting that the remaining clinical, vaccination-related, occupational, and epidemiological variables still contributed meaningful predictive information. These results support the relevance of severity stratification using routine surveillance data while emphasizing that future datasets should include more granular structural and healthcare-access variables to reduce reliance on broad geographic proxies.

Feature importance analysis identified seven key predictors of severity: county of residence, vaccination status, outbreak status, presence of other symptoms, occupation, cough, and conjunctivitis. All seven demonstrated statistically significant associations with disease severity, and six of seven showed significant geographic variation across counties, highlighting the complex interplay between epidemiological, socioeconomic, and clinical determinants of severe measles in this population.

The county of residence emerged as the single most important predictor, which warrants careful interpretation. The dataset empirically demonstrates statistically significant geographic variation in severity rates across the five counties of South-West Oltenia, with a nearly 20-fold gradient between the lowest (Gorj, 4.6%) and the highest (Vâlcea, 90.4%) severity proportions and identifies county of residence as the strongest single predictor in the model. It does not, however, contain direct individual-level measures of household income, educational attainment, distance to the nearest healthcare facility, family-physician density at the locality of residence, household composition or crowding, or ethnicity, so any attribution of the observed geographic gradient to specific structural determinants relies on regional-level statistics from external sources rather than on within-cohort measurement. With this distinction in mind, regional-level data describe South-West Oltenia as one of the most structurally vulnerable areas in the European Union: aggregate statistics report some of the lowest educational attainment levels (16–17% of the population aged 25–34 with tertiary education) [19], an at-risk-of-poverty rate 14.9 times higher than the Bucharest-Ilfov capital region, and severe material and social deprivation affecting nearly a quarter of the population [19]. The five counties, Dolj, Gorj, Mehedinți, Olt, and Vâlcea, share the same regional public-health administration centered in Craiova (Dolj) but are reported in regional and national statistics to differ in socioeconomic profile, healthcare infrastructure, and population structure. Dolj, which hosts the regional capital and the largest university medical center, has reportedly higher physician density and broader access to specialist care, while Mehedinți and Olt have been described as facing more pronounced primary-care shortages and limited hospital infrastructure. Vâlcea, where severe cases were concentrated in the present cohort, has been characterized in regional reports as combining mountainous terrain with dispersed rural settlements that may complicate access to healthcare. Gorj has been described as similarly semi-rural but with historically higher vaccination coverage linked to local public health engagement. These regional descriptions, drawn from external statistics rather than from the analyzed dataset, offer a plausible structural backdrop against which the observed inter-county variation in severity may be interpreted. The interpretation of county as a composite proxy for multiple structural determinants is theoretically coherent and consistent with the broader literature on measles in resource-constrained settings, but it remains untested at the individual level within the present study.

Beyond its interpretation as a composite proxy for unmeasured structural determinants, the dominance of county also has direct implications for the operational use of the framework. While clinicians evaluating an individual patient already know the patient’s county, the model’s role is not limited to individual clinical encounters: at the public-health level, the integrated geographic and individual-level risk estimates can support targeted resource allocation across counties, for example, prioritizing supplementary immunization activities, mobile clinical teams, or hospital surge capacity to counties with the highest predicted severity burden and can highlight where structural interventions are most needed. At the individual level, the framework provides a structured quantification of risk that combines geographic and clinical information in a single estimate, which can support less-experienced clinicians and high-throughput surveillance settings where informal integration of these factors is difficult. The strong contribution of county should therefore be interpreted not as a limitation of clinical utility but as an indication that future surveillance datasets need to include more granular structural and healthcare-access variables, vaccination coverage at the locality level, distance to nearest family physician, household crowding, and educational attainment of the household to enable individual-level risk stratification that is genuinely independent of geographic proxies.

Within this structural framing, the concentration of severe cases in Vâlcea may be compatible with the confluence of several factors documented in the broader literature to influence both disease transmission and medical response capacity. Lower educational attainment has been consistently associated with reduced vaccine acceptance, limited health literacy, and delayed recognition of disease severity [61]; financial constraints have been linked to limited access to healthcare and supportive care [23]; and overcrowded living conditions, more prevalent in disadvantaged communities, have been described as facilitating measles transmission given the virus’s high infectivity in closed environments [62]. We emphasize that none of these mechanisms can be directly tested in the present dataset, which contains no individual-level measures of income, education, household crowding, or distance to care; the discussion is therefore offered as a plausible interpretive context for the geographic pattern, not as a within-study causal finding. The empirical observation in the present cohort is the significant association between vaccination status and county of residence, which is consistent with the hypothesis that geographic clustering of under-vaccinated populations contributes to the observed disparities in severity. National data describe substantial regional variation in vaccination coverage across Romania, with rural and economically disadvantaged areas often experiencing the lowest rates [6]. The remaining structural mechanisms, household composition, viral inoculum, and similar, represent hypotheses generated by the geographic finding rather than results of the present analysis. The infectious disease management infrastructure in Dolj County, which serves as the regional referral center for the South-West Oltenia region, has demonstrated capacity to respond to rare and emerging zoonotic infections, as previously documented in the same geographic area [63].

Beyond structural opportunity-side barriers, the persistence of vaccination gaps in South-West Oltenia may also reflect motivational-side determinants of vaccine acceptance that are not fully explained by poverty, low educational attainment, or limited access to primary care alone. Vaccination in Romania is offered free of charge through family physicians and is actively recommended at the national level, yet refusal and delay continue at non-trivial rates even where access is in principle available. The healthism framework, articulated in recent international literature, offers a complementary explanatory layer for this residual gap [64]. As described by Kirbiš (2023), healthism captures the moralization of natural health practices and the valorization of bodily autonomy over biomedical intervention, alongside distrust in healthcare institutions, as motivational substrates for vaccine hesitancy that operate independently of socioeconomic disadvantage [64]. In the Romanian context, where mandatory vaccination policies were largely abandoned after 1989 and where multiple subsequent attempts at reinstatement have met sustained resistance, this resistance has been observed to operate partly through ideological and cultural channels rather than through access barriers alone, suggesting that healthism-type orientations may help account for why structural improvements alone, including outreach through family physicians and free-of-charge provision, have not closed the regional immunity gap. Importantly, these two mechanisms are not mutually exclusive: structural deprivation may shape the informational and cultural environments in which healthism orientations develop and circulate, while healthism orientations can in turn amplify the impact of structural barriers by reducing engagement with biomedical services even where these are available. Recognizing this dual mechanism situates the Romanian case within the broader international evidence base on vaccine refusal and indicates that interventions focused exclusively on access expansion may need to be paired with culturally informed communication strategies addressing trust, autonomy, and risk perception to achieve durable improvements in regional coverage.

The role of household clustering and community structure in measles transmission is well documented. Outbreaks tend to propagate through close-knit communities with lower vaccination coverage, where large households and frequent inter-household contact sustain transmission [65]. In the Romanian context, certain minority communities, including Roma populations, have historically experienced lower coverage due to healthcare access barriers, socioeconomic marginalization, and vaccine hesitancy [66,67]. Previous research from Western Romania identified Roma ethnicity as an independent risk factor for prolonged hospitalization in adult measles patients [34], consistent with broader health disparities affecting this population across Europe [68]; however, this variable was not available in the present surveillance dataset.

The significant associations between severity and both occupation and county of residence further underscore the importance of healthcare access. Romania faces substantial challenges in primary care provision, particularly in rural areas where the density of family physicians drops to 1 per 2500–3000 population compared with 1 per 1000 in urban settings [28]. Recent data indicate that 328 local communities currently lack a family physician, contributing to a nationwide shortfall of approximately 2200 family physicians [29]. Limited access to primary care reduces opportunities for routine vaccination, early diagnosis, and timely referral [31], and patients in underserved areas may present later with more advanced symptoms, increasing the likelihood of complications [32]. The finding that cough and conjunctivitis were both strongly associated with severity and county of residence may partially reflect geographic variation in healthcare-seeking behavior and the timing of clinical presentation. Conjunctivitis is a hallmark feature of measles, occurring in approximately 90% of cases as part of the classic prodromal triad of cough, coryza, and conjunctivitis [69]. The strong association with severity observed here (χ^2^ = 95.17, *p* < 0.00001) is clinically noteworthy, as ocular involvement may indicate more pronounced systemic inflammation. Measles-associated conjunctivitis ranges from mild bilateral injection to severe keratoconjunctivitis, which can progress to corneal ulceration and permanent visual impairment, particularly in vitamin A–deficient populations [70,71]. The WHO recognizes measles as a leading cause of preventable childhood blindness in developing countries, with ocular complications contributing substantially to long-term morbidity [72]. In resource-limited settings, conjunctivitis at initial presentation may warrant closer monitoring for progression to more serious ocular and systemic complications. Beyond clinical management, prevention through vaccination remains the most effective strategy to avoid such outcomes altogether.

The absence of mandatory vaccination requirements linked to school enrollment represents a significant policy gap relative to other European countries [10]. Italy, France, and Germany each introduced mandatory vaccination following measles outbreaks, often achieving rapid increases in coverage [73]. Hungary, which maintained mandatory policies throughout the post-communist period, reached 99% coverage and has been described as an “island of safety” in Europe [11]. The contrast with Romania’s recommended but non-mandatory approach underscores the potential role of policy interventions in closing the immunity gaps that sustain recurrent epidemics.

The operational definition of severe cases employed here departed from the WHO criteria, which encompass a broader spectrum of complications, including severe diarrhea with dehydration, acute encephalitis, acute disseminated encephalomyelitis, and subacute sclerosing panencephalitis [52]. The absence of documented diarrhea, encephalitis, or severe neurological manifestations in the regional dataset may reflect true epidemiological patterns but could also stem from underreporting or systematic gaps in data collection, as noted in other contexts [54]. International data suggest that approximately 30% of measles cases experience at least one complication, with pneumonia and diarrhea representing the most common causes of morbidity and mortality [74], making the complete absence of reported diarrhea in this region a conspicuous gap. The WHO estimates that complications are most frequent in children under 5 and adults over 30 and more likely in malnourished individuals, those with vitamin A deficiency, or those with compromised immune systems [1]. The strong associations between respiratory symptoms and disease severity observed here align with international evidence that pneumonia is the leading cause of measles-related mortality [75]. Studies from the United States have reported that hospitalized measles patients experienced elevated rates of gastrointestinal, hematologic, infectious, neurologic, ophthalmologic, pulmonary, and renal complications, with encephalitis showing the strongest association with measles diagnosis [35]; however, no neurological complications were reported in our region. A study from Western Romania identified chronic lung disease, liver damage, Roma ethnicity, duration since last MMR dose, elevated inflammatory markers, and bilateral pulmonary consolidation as independent predictors of prolonged hospitalization [34].

The superior performance of RF over LR is consistent with the growing body of evidence supporting ensemble machine learning techniques in clinical prediction [76,77]. The markedly higher AUC (0.87 vs. 0.80) indicates enhanced capacity to capture complex, non-linear interactions among clinical and demographic variables, relationships that traditional linear approaches may not adequately model [78]. From a clinical standpoint, the two models have approximately equivalent sensitivities (approximately 0.81), meaning each correctly identified approximately 81% of true severe cases, which is particularly important in public health applications, where missed severe cases could delay intervention. However, the higher specificity of RF (0.87 vs. 0.84) suggests fewer false-positive classifications, potentially helping avoid unnecessary diagnostic procedures or resource misallocation [79]. The higher F1-score and predictive values further indicate that RF maintains a more favorable precision–recall balance, particularly when severe cases constitute a smaller fraction of the study population. This robustness in real-world clinical datasets supports the potential utility of RF-based models in decision support systems for measles severity prediction [80]. Vaccination status, identified as the second most important predictor, is a directly modifiable risk factor amenable to targeted immunization campaigns and catch-up programs [8]. The identification of geographic clustering of severe cases and under-vaccination can inform spatial targeting of interventions to communities at highest risk. Although the present analysis focuses on measles surveillance data, the proposed framework may inform the development of scalable digital risk stratification approaches applicable to other sustained public health burdens in resource-limited contexts.

Beyond aggregate performance metrics, the clinical implications of misclassification warrant explicit consideration. With an overall accuracy of 0.84 for the RF model, approximately 16% of cases were misclassified per cross-validation iteration, corresponding to roughly 100 of 624 cases when extrapolated across the full dataset. These errors are not symmetrical: with a sensitivity of approximately 0.81, an estimated 19% of true severe cases were not flagged by the model (false negatives), while a specificity of 0.87 means that approximately 13% of non-severe cases received a severity flag they did not warrant (false positives). False negatives are clinically more consequential, as they may delay escalation of care for patients with evolving complications; false positives, while less harmful, may generate unnecessary hospital referrals or resource utilization. Both error types are therefore explicitly acknowledged as inherent properties of probabilistic classification on routinely collected surveillance data, and neither undermines the role of the model as a triage-support tool. The model is intended to operate upstream of clinical evaluation, prioritizing cases for closer review rather than replacing the physician’s diagnostic role. All flagged and unflagged cases continue to require direct clinical assessment, and any operational deployment of such a tool would need to incorporate physician oversight, periodic recalibration, and integration with established clinical pathways.

Several limitations should be acknowledged. The retrospective design and reliance on routinely collected surveillance data introduce potential biases related to completeness, accuracy, and consistency of clinical documentation across facilities [81]. The absence of certain complications included in the WHO definition may reflect true patterns but could also result from underreporting or from systematic data-collection gaps. The complete absence of diarrhea, encephalitis, acute disseminated encephalomyelitis, subacute sclerosing panencephalitis, and otitis media across all 624 cases is a notable feature of the dataset that warrants careful consideration. Three non-exclusive scenarios may account for this finding, and the available data do not allow them to be empirically distinguished. First, these complications may have been genuinely absent in the cohort, which would itself represent an unexplained epidemiological observation given their well-documented occurrence in measles cohorts internationally. Second, they may have occurred clinically but been systematically underreported, with the affected cases not having the corresponding fields completed in the regional surveillance system. Third, the documentation of complications may have been non-standardized across reporting facilities, with the same clinical event recorded under different fields, in narrative free text, or not at all, depending on local practice. Each scenario carries distinct analytic implications. Under the first, the operational severity definition is unbiased but more restrictive than the full WHO criteria. Under the second and third, the severity label may be both biased and incomplete, with cases having unreported neurological or gastrointestinal complications misclassified as non-severe, yielding conservative performance estimates relative to a fully ascertained dataset. Importantly, this issue extends beyond the severity label: if reporting completeness varies by county, reporting facility, or physician training, then predictor distributions and the conditional structure linking predictors to severity may be differentially affected across geographic strata. This would tend to inflate the apparent predictive contribution of county of residence because county would partially capture differences in reporting practice in addition to genuine epidemiological gradients. Generalizability is therefore constrained: deployment in regions with more complete or more standardized complication reporting may yield different severity proportions, different predictor importances, and different absolute performance. We recommend that future prospective regional surveillance datasets adopt standardized complication fields with mandatory capture of WHO-recognized severe manifestations, accompanied by individual-level structural variables and protocols to minimize facility-level documentation heterogeneity, to allow direct empirical resolution of the scenarios outlined above.

The moderate sample size, while sufficient for the approaches employed, may limit generalizability. The cross-validation framework mitigates overfitting, but external validation on independent datasets would strengthen confidence in the reported estimates [82]. The study was restricted to the South-West Oltenia region, which may not be representative of measles epidemiology elsewhere in Romania or in countries with different healthcare systems, vaccination policies, and socioeconomic conditions. The significant variation observed across counties within this single region underscores the importance of local context. Several specific data limitations also constrained the analysis. Certain clinical predictors documented in the broader measles literature were unavailable in the surveillance dataset, including nutritional status, vitamin A supplementation, underlying comorbidities, and timing of healthcare presentation, all of which have been identified as determinants of severity in other settings and whose absence may have limited model performance [83]. The dataset also contained no individual-level structural variables, including household income, educational attainment, distance to the nearest healthcare facility, family-physician density at the locality of residence, household composition or crowding, and ethnicity. This second class of omissions has two analytic consequences: it constrains the structural interpretation of the county effect to a hypothesis-generating account based on external regional statistics rather than within-cohort measurement, and it likely contributes to the strong predictive weight of county of residence, which in part absorbs the variance these missing variables would otherwise have captured. Future iterations of regional surveillance should consider integrating both clinical and structural variables at the case level to allow individual-level risk stratification that is genuinely independent of geographic proxies and to enable direct empirical testing of the structural mechanisms hypothesized here. While the models demonstrated good discriminative ability, they do not establish causal relationships. The associations identified should be treated as hypothesis-generating findings warranting further investigation through appropriately designed epidemiological studies.

Finally, the model is not an error-proof diagnostic system and is not intended to function as a standalone clinical decision-maker. With approximately 16–18% of cases misclassified, the model carries inherent uncertainty that must be addressed through physician oversight, clinical correlation, and integration into existing surveillance and care pathways. The reported performance should be interpreted as the achievable upper bound of a probabilistic classifier trained on routinely collected, partially incomplete surveillance data, rather than as a benchmark for autonomous clinical use.

## 5. Conclusions

The South-West Oltenia region faces a difficult reality: low educational attainment, elevated poverty, and uneven healthcare access are deeply entrenched structural problems unlikely to resolve in the near term. Romania’s vaccination policy, which recommends but does not mandate immunization, similarly shows limited prospects for immediate reform. As long as these conditions persist, measles transmission and its severe consequences will continue to disproportionately affect the most vulnerable communities in this region.

Against this backdrop, the present study shows that machine learning, specifically Random Forest, can meaningfully extract clinically and geographically structured patterns from routinely collected surveillance data and translate them into a triage-support tool applicable to future cases entering the same surveillance system. Although the operational definition of severe cases was derived from complication fields already documented in the regional surveillance database, the model’s contribution lies in identifying which combinations of demographic, clinical, and geographic predictors are most strongly aligned with this severity label, and in providing a reproducible classification framework that can be applied prospectively to newly reported cases before complete complication data are available. The RF model outperformed conventional Logistic Regression across most performance metrics (accuracy 0.84 vs. 0.82; AUC 0.87 vs. 0.80; *p* ≤ 0.001), and the feature importance analysis pointed to county of residence, vaccination status, outbreak status, cough, conjunctivitis, occupation, and other symptoms as the strongest predictors aligned with severe presentations. That county of residence emerged as the single most influential variable is perhaps the most telling finding: it suggests that where a patient lives and, by extension, what healthcare, vaccination infrastructure, and socioeconomic conditions that location affords, is associated with severity at least as strongly as any individual clinical feature.

More broadly, these results suggest that machine learning retains practical value in infectious disease surveillance even when the data infrastructure does not support reliable outbreak prediction. Where fragmented reporting systems and limited regional integration make transmission forecasting impractical, severity classification offers a more achievable goal, one that can still inform early identification of high-risk patients, guide resource allocation, and support targeted public health responses. Whether this approach can be extended to other vaccine-preventable diseases or adapted for use in other resource-limited settings remains an open question, but the framework presented here offers a reasonable starting point.

In all cases, predictions generated by such models must be interpreted within the broader clinical and epidemiological context and should support, not replace, physician judgment.

It should be emphasized that the reported performance metrics characterize discrimination within the surveillance population from which the model was trained, and the framework is intended to support severity stratification among cases already entering the public-health surveillance system rather than to estimate measles severity at the general-population level.

## Figures and Tables

**Table 1:** Summary of variables included in the machine learning analysis.

Category	Variable	Type
Demographic and Socio-geographic	Age	Continuous (years)
	Gender	Binary (male/female)
	County	Categorical (5 levels)
	Environment	Binary (urban/rural)
	Occupation	Categorical (5 levels)
Healthcare Access and Hospitalization	Family doctor registered	Binary (yes/no)
	Hospitalization	Binary (yes/no)
	Length of hospital stay	Continuous (days)
Clinical Presentation and Complications	Fever	Binary (yes/no)
	Rash	Binary (yes/no)
	Coryza	Binary (yes/no)
	Cough	Binary (yes/no)
	Conjunctivitis	Binary (yes/no)
	Arthritis	Binary (yes/no)
	Arthralgia	Binary (yes/no)
	Lymphadenopathy	Binary (yes/no)
	Other symptoms	Binary (yes/no)
	Clinical severity criteria met	Binary (yes/no)
	No complications reported	Binary (yes/no)
	Pneumonia	Binary (yes/no)
	Thrombocytopenia	Binary (yes/no)
Vaccination and Immunological History	Vaccination status	Categorical (4 levels)
	History of measles infection	Binary (yes/no)
	Not eligible for vaccination	Binary (yes/no)
Epidemiological Exposure	Contact with a confirmed case	Binary (yes/no)
	Recent travel history	Binary (yes/no)
	Association with a known outbreak	Binary (yes/no)

**Table 2:** Key performance metrics used for model assessment.

Metric	Formula
Accuracy	(TP + TN)/(TP + TN + FP + FN)
Sensitivity	TP/(TP + FN)
Specificity	TN/(TN + FP)
Positive Predictive Value (PPV)	TP/(TP + FP)
Negative Predictive Value (NPV)	TN/(TN + FN)
F1-score	2 × (Precision × Recall)/(Precision + Recall)
Odds ratio (OR)	(TP × TN)/(FP × FN)

**Table 3:** Distribution of Measles Cases by County and Severity Category (South-West Oltenia, 2023–2024).

County	Severe, n (%)	Non-Severe, n (%)	Total, n (%)	Severity Rate (%)
Vâlcea (VL)	281 (90.4)	30 (9.6)	311 (49.8)	90.4
Dolj (DJ)	36 (23.1)	120 (76.9)	156 (25.0)	23.1
Olt (OT)	19 (25.3)	56 (74.7)	75 (12.0)	25.3
Gorj (GJ)	3 (4.6)	62 (95.4)	65 (10.4)	4.6
Mehedinți (MH)	4 (23.5)	13 (76.5)	17 (2.7)	23.5
**Total**	**343 (55.0)**	**281 (45.0)**	**624 (100.0)**	**55.0**

**Table 4:** Model performance assessment. Values are expressed as mean ± standard deviation. Statistical significance was assessed using paired Student’s *t*-tests; *p* < 0.05 was considered statistically significant (bold).

	Accuracy	AUC	Sensitivity	Specificity	PPV	NPV	F1
RF	0.84 ± 0.04	0.87 ± 0.05	0.81 ± 0.06	0.87 ± 0.06	0.89 ± 0.05	0.80 ± 0.06	0.84 ± 0.04
LR	0.82 ± 0.05	0.80 ± 0.06	0.81 ± 0.06	0.84 ± 0.07	0.86 ± 0.05	0.79 ± 0.06	0.83 ± 0.04
*p*, *t* Test	**<0.001**	**<0.001**	0.328	**<0.001**	**<0.001**	**0.001**	**<0.001**

**Table 5:** Model performance assessment after exclusion of county as a predictor variable. Values are expressed as mean ± standard deviation. Statistical significance was assessed using paired Student’s *t*-tests; *p* < 0.05 was considered statistically significant (bold).

	Accuracy	AUC	Sensitivity	Specificity	PPV	NPV	F1
RF	0.68 ± 0.03	0.73 ± 0.03	0.72 ± 0.05	0.64 ± 0.05	0.69 ± 0.02	0.66 ± 0.03	0.70 ± 0.03
LR	0.66 ± 0.03	0.68 ± 0.03	0.68 ± 0.04	0.63 ± 0.05	0.68 ± 0.03	0.63 ± 0.03	0.68 ± 0.03
*p*, *t* Test	**<0.001**	**<0.001**	**<0.001**	0.302	**<0.001**	0.001	**<0.001**

## Data Availability

The data can be obtained with a written request to the Regional Center for Public Health Surveillance Craiova (Centrul Regional de Sănătate Publică Craiova).

## Abbreviations

The following abbreviations are used in this manuscript:

AI	Artificial Intelligence
AUC	Area Under the Curve
AUC-ROC	Area Under the Receiver Operating Characteristic Curve
CDC	Centers for Disease Control and Prevention
DJ	Dolj County
DSP	Direcția de Sănătate Publică (Public Health Directorate)
EU	European Union
FN	False Negative
FP	False Positive
GJ	Gorj County
LR	Logistic Regression
MH	Mehedinți County
ML	Machine Learning
MMR	Measles, Mumps, and Rubella
NPV	Negative Predictive Value
OT	Olt County
PPV	Positive Predictive Value (Precision)
RF	Random Forest
ROC	Receiver Operating Characteristic
RS	Random Subspace
TN	True Negative
TP	True Positive
VL	Vâlcea County
WHO	World Health Organization
